# Microstructure changes in whiter matter relate to cognitive impairment in Wilson’s disease

**DOI:** 10.1042/BSR20181651

**Published:** 2019-03-15

**Authors:** Ting Dong, Wen-ming Yang, Ming-cai Wu, Juan Zhang, Peng Huang, Chun-sheng Xu, An-qin Wang, Chun-jun Kuang, Zhi-ling Gao

**Affiliations:** 1 Department of Neurology, The First Affiliated Hospital of Anhui University of Chinese Medicine, Hefei, Anhui, China; 2 Anhui Province Key Laboratory of Active Biological Macro-molecules, Wannan Medical College, Wuhu, Anhui, China; 3 Department of Medical Imaging, The First Affiliated Hospital of Anhui University of Chinese Medicine, Hefei, Anhui, China; 4 Department of Intensive Care Units, The First Affiliated Hospital of Anhui University of Chinese Medicine, Hefei, Anhui, China

**Keywords:** adolescent, cognitive impairment, fractional anisotropy, Wilson’s disease

## Abstract

**Purpose:** Wilson’s disease (WD) is a genetic disorder of copper metabolism with pathological copper accumulation in the brain. The purpose of the present study was to evaluate the relationship between the damaged white matter and the impaired cognitive function in WD patients. **Materials and methods:** Thirty WD adolescents and thirty age- and sex-matched healthy controls (HC) were enrolled. All subjects had received brain MRI, including conventional and diffusion-tensor imaging (DTI) scans. The DTI parameter of fractional anisotropy (FA) was calculated by diffusion kurtosis estimator software. The *t* test was used to compare the differences between two groups. The correlation between cognitive function and whiter matter disorders were analyzed by linear regression. The results of FA parameter and MD parameter intergroup analysis were both corrected with False Discovery Rate (FDR) simulations by SPSS. **Results:** WD adolescents showed significantly lower scores of time-based prospective memory (TBPM) and verbal fluency test (VFT) compared with HC. We found significantly higher FA in the right thalamus, right lentiform nucleus, left thalamus, left lentiform nucleus, and brain stem in WD adolescents. Besides, WD adolescents exhibited significantly lower FA in right cerebellum and cingulum and left middle frontal lobe compared with controls (*P*<0.05). There were significantly negative correlations between FA in bilateral lentiform and thalamus and cognitive impairment in WD adolescents (*P*<0.05). **Conclusion:** The whiter matter of WD adolescents was impaired and mainly distributed in subcortical brain regions. The impaired cognitive function was affected by the damaged whiter matter. The present study may be helpful for recognition and understanding of WD.

## Introduction

Wilson’s disease (WD), known as progressive hepatolenticular degeneration, is a genetic disorder commonly supposed due to a mutation of gene *ATP7B* responsible for copper metabolism, and usually occurs in children. WD may lead to severe disability and death [1,2]. Routine MRI is usually used in its diagnosis, that exhibits symmetrical T2 hyperintensity or mixed intensity in caudate nuclei, thalami, pons putamina, or globi pallida [3–6]. However, some types of WD do not have significant signs in the MRI method, and thus more accurate quantitative measurement could be applied, such as diffusion-weighted imaging (DWI) and diffusion-tensor imaging (DTI) [7,8]. Fractional anisotropy (FA) is the main parameter of DTI, which is usually applied to identify the microstructural abnormalities in whiter matter. In previous studies, DTI has been applied to assess the microstructure of thalamus and evaluate diffusion abnormalities in the white matter regions in WD patients [9].

Cognitive impairment of WD patients was known to be associated with the low educational level and MRI hyperintensity in the basal ganglia nucleus [10,11]. The correlation between decreased FA in white matter and cognitive impairment was also reported in different brain diseases [12–14]. But little is known about the relationship(s) between the abnormal FA and cognitive impairment in WD patients. Therefore, the current study was to evaluate the microstructural abnormities in whiter matter with DTI technique and further assess how such abnormities affect cognitive function in WD patients by exploring the correlation between cognitive function and brain FA changes.

## Materials and methods

### Patients

The present study was approved by the First Affiliated Hospital of Anhui University of Chinese Medicine (AUCH) Ethics Committee and was conducted in accordance with the Declaration of Helsinki. The informed consents were obtained from all patients before enrollment.

WD adolescents who were hospitalized in the First Affiliated Hospital of ACUM from April 2014 to December 2016 were recruited in the present study. The diagnostic criteria of the WD group were as follows: (i) with neurological symptoms and psychiatric symptoms; (ii) with hepatic symptoms; (iii) with corneal Kayser–Fleischer ring; and (iv) other findings from microscopic examination or lab examination: serum copper oxidase level < 0.2 mg/ml and/or serum copper-blue protein level < 200 mg/l, 24-h urinary copper excretion > 100 μg (1.56 μmol), hepatic copper dry weight ≥ 250 μg/gm, hematuria, microalbuminuria, renal tubular acidosis, osteoarthrosis etc. [15,16].

The inclusion criteria were: (i) between 14 and 40 years and with ≥5 years of education; (ii) without intelligence deficits (IQ > 80 score); (iii) one or two grades of Modified Goldstein’s Degree [17]**;** (iv) the Chinese version of Unified WD Rating Scale for neurological function < 35 score [18,19]; (v) right-handedness; (vi) without color blindness, blindness, or deafness; (vii) without cognition impairment caused by other diseases or drugs; (viii) no drug abuse history; and (ix) Mini-Mental State Examination (MMSE) score > 23.

For healthy control (HC) group, the inclusion criteria were: (i) between 14 and 40 years and with ≥5 years of education; (ii) without intelligence deficits (IQ > 80 score); (iii) right-handedness; (iv) no color blindness, blindness, or deafness; (v) no cognitive impairment caused by other diseases or drugs; (vi) no history of neurological and mental disease; (vii) no drug abuse history; (viii) no family history of mental illness; and (ix) MMSE score > 23.

### MRI protocol

All these included patients had received brain MRI, including conventional and DTI scan. Healthy volunteers received only DTI scan. A Signa VH/i 3.0 T MR imaging system (General Electric Medical Systems, Milwaukee, WI) was used with 8-channel high-resolution radio-frequency head coil. For conventional MRI, the sequences included T2 Flair (repetition time = 9000 ms, echo time = 124 ms, flip angle = 111°, matrix size = 256 × 256, field of view = 250 × 250 mm, layer thickness = 5 mm, no spacing and scanning 20 layers), T1-3D BRAVO (repetition time = 8.2 ms, echo time = 3.2 ms, flip angle = 12°, matrix size = 256 × 256, field of view = 256 × 256 mm, layer thickness = 1 mm, no spacing and scanning 166 layers). DTI was performed using the echo-planar imaging (EPI) sequence (repetition time = 6000 ms, echo time = minimum, matrix size = 128 × 128, field of view = 256 × 256 mm, layer thickness = 3 mm, no spacing and scanning 50 layers, diffusion sensitivity coefficient b = 0 s/mm^2^ and 1000 s/mm^2^, 64 direction).

### Data analysis

A voxel-based TBSS approach was used for the group analysis of DTI data. DTI datasets were processed with the Functional MRI of the brain (FMRIB) Software Library (FSL) software package (www.fmrib.ox.ac.uk/fsl). Preprocessing included Eddy current and motion correction and brain-tissue extraction. After preprocessing, DTI images were averaged and concatenated, and a diffusion tensor model was fitted at each voxel to generate FA maps. [20,21]. Images were warped to the Montreal Neurological Institute (MNI) 152 template, available as a standard T1 dataset in the FSL software package. TBSS was run with FA maps to create the ‘skeleton’, which represented the center of all fiber bundles common to all subjects. Intergroup analysis was performed with test to investigate variation of microstructure in the brain between the patients of WD and control group. The results of FA parameter and MD parameter intergroup analysis were both corrected with False Discovery Rate (FDR) simulations.

### Evaluation of cognitive function

The MMSE, the time-based (TBPM) and event-based prospective memory (EBPM) tests, as well as digit span (DS) and verbal fluency test (VFT), were performed as previous studies mentioned [22–25].

### Correlation analysis

Categorical variables were expressed as number and percentage. All continuous variables were expressed as mean ± S.D. (normal distribution) or median and interquartile range (non-normal distribution) based on the results of the Kolmogorov–Smirnoff test. The *t*test (normal distribution) or Mann–Whitney U test (non-normal distribution) was used to compare WD and control individuals. The Spearman coefficient was used to assess the correlation between FA and cognitive function amongst WD adolescents. A *P*-value less than 0.05 was considered statistically significant in all analyses.

## Results

### Patient characteristics

A total of 30 WD adolescents and 30 HC were enrolled in the present study. Compared with controls, WD patients showed longer T1, longer T2 in bilateral basal ganglia and brain stem. There were no significant differences between WD and HC groups in age, gender, and education levels (*P*>0.05, Table 1). The mean WD duration of the patients was 2.64 ± 0.88 years (Table 1).

**Table 1:** Characteristics of two groups of participants

	Control (*n*=30)	WD (*n*=30)	*P*
**Age (years)**	16.97 ± 1.16	16.90 ± 2.04	0.877
**Male/Female**	15 (50%)/15 (50%)	15 (50%)/15 (50%)	1.000
**Education (years)**	8 (8–8)	8 (8–8)	1.000
**Duration of disease (years)**	-	2.64 ± 0.88	-

### FA changes in WD patients

**Figure 1:**
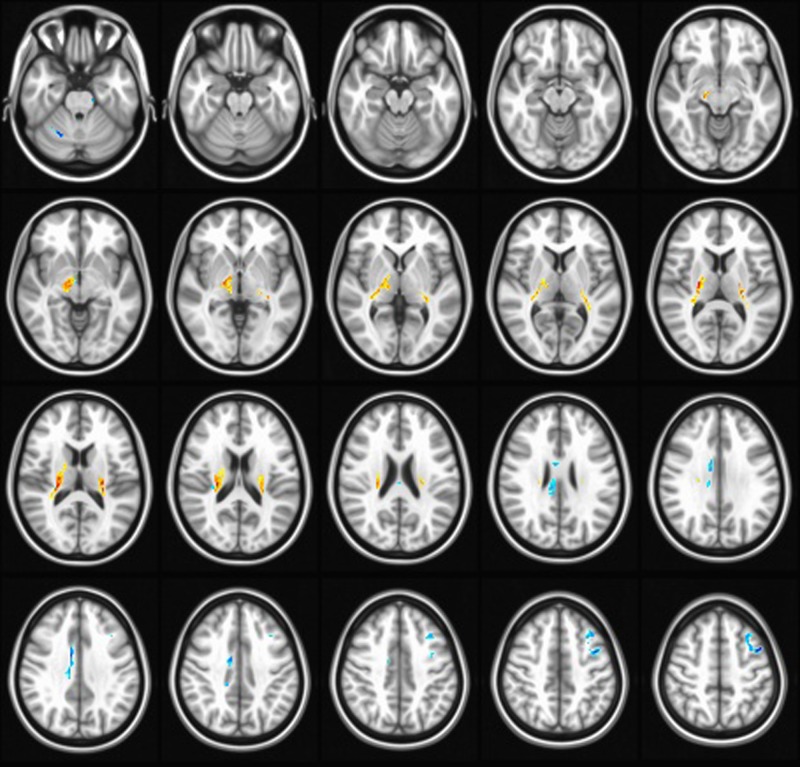
FA parameter differences of brain regions between patients and controls (FDR simulation, *P*=0.001, α = 0.05, cluster size = 326) Compared with the HC, patients showed increased FA in in bilateral thalamus, bilateral lentiform nucleus, and decreased FA in right cerebellum and cingulum, and left middle frontal lobe.

For cognitive function evaluation (Table 2), the WD patients had scores of EBPM, MMSE, and DS similar to controls (*P*>0.05), while they exhibited significantly decreased TBPM (*P*<0.001) and VFT (*P*=0.019) scores. For MRI evaluation (Table 3 and Figure 1), WD patients showed increased FA values in bilateral thalamus, bilateral lentiform nucleus, compared with HC (Figure 1). Simultaneously, FA in right cerebellum and cingulum, and left middle frontal lobe were significantly lower in WD patients compared with the HC (Figure 1).

**Table 2:** Comparison of cognitive function between WD and HC groups

	Control (*n*=30)	WD (*n*=30)	*P*
**EBPM**	6 (5–6)	5 (5–6)	0.350
**TBPM**	6 (5.75–6)	3 (2–4)	<0.001
**MMSE**	28 (28–29)	28 (27–28)	0.069
**DS**	7 (6.75–8)	7 (5.75–8)	0.195
**VFT**	9 (8–9)	8 (7–9)	0.019

**Table 3:** Comparison of the FA in different brain areas between WD and HC groups

Areas	Control (*n*=30)	WD (*n*=30)	*P*
**Right thalamus**	0.137 ± 0.020	0.200 ± 0.048	<0.001
**Right lentiform nucleus**	0.132 ± 0.011	0.194 ± 0.044	<0.001
**Left thalamus**	0.132 ± 0.014	0.199 ± 0.055	<0.001
**Left lentiform nucleus**	0.130 ± 0.011	0.194 ± 0.047	<0.001
**Right head of caudate nucleus**	0.200 ± 0.037	0.126 ± 0.024	<0.001
**Brain stem**	0.292 ± 0.018	0.352 ± 0.053	<0.001
**White matter**	0.282 ± 0.060	0.210 ± 0.042	<0.001

### Correlation between FA and cognitive function

**Figure 2:**
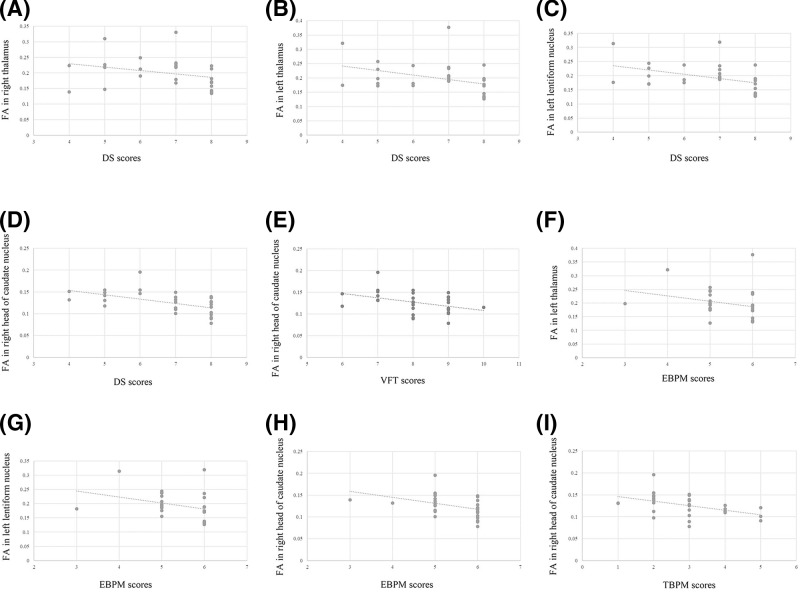
The correlation of FA and cognitive function in WD patients The DS score and FA in the right thalamus (**A**), left thalamus (**B**), left lentiform nucleus (**C**) and right head of caudate nucleus (**D**); the FT score and FA in right head of caudate nucleus (**E**); the EBPM score and FA in left thalamus (**F**), left lentiform nucleus (**G**) and right head of caudate nucleus (**H**); the TBPM score and FA in right head of caudate nucleus (**I**).

As shown in Table 4, no significant correlation between FA and cognitive function was found in the control group, based on EBPM, TBPM, MMSE, DS, and VFT (*P*>0.05 or/and Pearson r < 0.4). However, a significant correlation between FA and cognitive function was found in WD patients, based on EBPM, TBPM, DS, and VFT (Table 5 and Figure 2). We observed significantly negative correlation between the EBPM score and FA in left thalamus (r = −0.424, *P*=0.020), left lentiform nucleus (r = −0.447, *P*=0.013), and between the DS score and FA in right thalamus (r = −0.424, *P*=0.020), left thalamus (r = −0.421, *P*=0.021), left lentiform nucleus (r = −0.435, *P*=0.016).

**Table 4:** Correlation of FA and cognitive function in HC group

	EBPM	TBPM	MMSE	DS	VFT
**Right thalamus**	0.134	−0.168	−0.079	−0.328	0.073
**Right lentiform nucleus**	0.079	−0.077	0.163	−0.183	0.099
**Left thalamus**	−0.399*	−0.141	0.283	−0.146	0.236
**Left lentiform nucleus**	−0.291	0.023	0.287	−0.083	0.138
**Right head of caudate nucleus**	-0.166	−0.241	−0.045	−0.213	0.247
**Brain stem**	0.312	0.059	0.144	0.106	0.000
**White matter**	0.083	−0.159	−0.005	0.207	0.059

* *P*=0.029.

**Table 5:** Correlation of FA and cognitive function in WD group

	EBPM	TBPM	MMSE	DS	VFT
**Right thalamus**	−0.211	−0.102	−0.213	−0.424*	−0.111
**Right lentiform nucleus**	−0.163	−0.122	0.173	−0.376*	−0.040
**Left thalamus**	−0.424*	−0.285	0.220	−0.421*	0.103
**Left lentiform nucleus**	−0.447*	−0.296	0.172	−0.435*	0.052
**Right head of caudate nucleus**	−0.490^†^	−0.510^†^	0.081	−0.590^†^	−0.408*
**Brain stem**	−0.100	−0.072	−0.008	−0.354	−0.218
**White matter**	0.067	0.223	0.003	0.110	0.116

*P<0.05. ^†^*P*<0.01.

## Discussion

WD is an autosomal recessive disorder of copper metabolism. The recognized mechanism is the dysfunction of a copper-transporting ATP7B, which causes aberrant copper accumulation. However, the changes in diffusion images and the cognition damage have been seldom analyzed. Theoretically, WD involves pathological changes in a cerebello–thalamo–cortical network. In the present study, we found verbal intelligence ability and memory speed in WD adolescents was markedly deficient, in comparison with heathy controls based on the tests including TBPM and VFT. MRI results showed significantly enhanced FA in right thalamus, bilateral lentiform nucleus, bilateral thalamus, as well as reduced FA in right cerebellum and cingulum and left middle frontal lobe in WD adolescents. FA is the main parameter of DTI, which is usually applied to identify the microstructural abnormalities in whiter matter. FA variation strongly implied the microstructural changes in whiter matter [26–29]. Interestingly, we found a correlation between FA and EBPM and DS in WD adolescents, but WD and healthy individuals had similar EBPM and DS scores. This outcome suggests that the indicators like EBPM and DS scores could not sufficiently reflect the pathological characteristics, and may be impacted much later during WD development, compared with TBPM and VFT scores, in despite that they may be truly influenced by WD and enhanced FA. The negative correlation between FA in right head of caudate nucleus and TBPM/VFT score suggested the relationship of cognitive impairment (in the aspects of verbal intelligence ability and memory speed) and FA in right head of caudate nucleus. This is the first report noticing the FA variation features in WD and the its correlation with cognitive impairment in WD. Our observation innovatively suggests that the impaired cognitive function was affected by the damaged whiter matter, and the conclusion may be helpful for recognition and understanding of WD.

There exists some indirect supportive evidences. For example, WD adolescents had decreased FA in white matter [9]. In caudate nucleus, decreased signal intensity in WD patients by MRI was reported [30], which was consistent with our findings. Currently, it has been seldom reported about the MR spectroscopy diffusion MRI in WD patients. Although no indirect evidence in previous studies support the results of increased FA in right thalamus, right lentiform nucleus, left thalamus, left lentiform nucleus, and brain stem, the lesions in thalamus, lentiform nucleus, and brain stem have been seen repeatedly [30–32]. We here provided new evidences using the MRI signs. In consistency, other advanced MRI application except DWI and DTI, such as susceptibility-weighted imaging (SWI), have indicated decreased corrected phase values in WD patients [33].

There have been limited studies that applied DTI to assess the FA variation in WD patients. Taly et al. observed decreased FA in the frontal and occipital white matter, bilateral internal capsules, midbrain, and pons in WD patients [34]. Chen et al. reported significantly different FA values in thalamus between WD and healthy population [35]. They and Zhang et al. found increased FA in the bilateral head of the caudate nucleus, lenticular nucleus, ventral thalamus, substantia nigra, red nucleus, right dentate nucleus, and decreased in the mediodorsal thalamus and extensive white matter [36]. Some of their findings were proved by our study, e.g. the changes in caudate nucleus and white matter.

Previous studies have noticed significant correlation between FA in white matter and cognitive impairment [12–14], but this was not found in WD adolescents in our study. Many studies have showed the correlation of cognitive impairment with white matter damage based on the diffusion MRI [37–39]. We found no association between FA in white matter and verbal intelligence ability or memory speed at present. However, the negative correlation of FA in right head of caudate nucleus with cognitive impairment was determined in the present study. Cognitive impairment is known to be associated with the caudate nucleus lesion in published articles [40,41]. Moreover, the decreased FA in right head of caudate nucleus may be associated with deficient verbal intelligence ability and memory speed in WD adolescents, resulting in the significantly negative correlation between FA in right head of caudate nucleus with TBPM and VFT scores in the present study. A previous study reported that the caudate nucleus volume was associated with the dopamine receptor D2 (DRD2) Taq I genetic polymorphism in the memory impaired subjects [42]. DRD2 plays an important role in memory processes [43] and is associated with verbal intelligence quotient [44]. Thus, the abnormal FA in right head of caudate nucleus may be associated with cognitive function of WD adolescents via the mechanism of DRD2. The cognitive function of WD adolescents should be further evaluated by additional measurements in order to verify these above speculations.

Also, cognitive decline might be associated with the atrophy of cortices. The voxel-based morphometry study showed that the structural alterations in gray matter and white matter may implied a cognitive decline [45,46]. Further studies are needed to investigate the association between cognitive impairment with voxel-based morphometry, MD, or other factors in WD adolescents.

## Conclusion

WD adolescents had cognitive impairment, indicated by TBPM and VFT, and abnormal FA in several brain regions. There exists significantly negative correlation between FA in bilateral lentiform and thalamus and cognitive impairment in WD adolescents. This indicates that the impaired cognitive function was affected by the damaged whiter matter. The present study may be helpful for recognition and understanding of WD.

## Abbreviations

DRD2: dopamine receptor D2
DS: digit span
DTI: diffusion-tensor imaging
DWI: diffusion-weighted imaging
EBPM: event-based prospective memory
FA: fractional anisotropy
FSL: Functional MRI of the brain (FMRIB) Software Library
HC: healthy control
MMSE: Mini-Mental State Examination
TBPM: time-based prospective memory
VFT: verbal fluency test
WD: Wilson’s disease
